# Differential General Anesthetic Effects on Microglial Cytokine Expression

**DOI:** 10.1371/journal.pone.0052887

**Published:** 2013-01-31

**Authors:** Xuefei Ye, Qingquan Lian, Maryellen F. Eckenhoff, Roderic G. Eckenhoff, Jonathan Z. Pan

**Affiliations:** 1 Department of Anesthesiology and Critical Care, Perelman School of Medicine at the University of Pennsylvania, Philadelphia, Pennsylvania, United States of America; 2 Department of Anesthesiology, The Second Affiliated Hospital of Wenzhou Medical College, Wenzhou, Zhejiang, China; Massachusetts General Hospital, United States of America

## Abstract

Post-operative cognitive dysfunction has been widely observed, especially in older patients. An association of post-operative cognitive dysfunction with the neurodegenerative diseases, such as Alzheimer's disease, has been suggested. Neuroinflammation contributes to Alzheimer pathology, through elevated pro-inflammatory cytokines and microglial activation in the CNS leading to neuronal damage, synaptic disruption and ultimately cognitive dysfunction. We compare the effects of three different, clinically-used, anesthetics on microglial activation with, and without, the prototypical inflammatory trigger, lipopolysaccharide (LPS). Microglial BV-2 cell cultures were first exposed to isoflurane, sevoflurane (each at 2 concentrations) or propofol for 6 h, and cytokine levels measured in lysates and media. The same experiments were repeated after 1 h LPS pre-treatment. We found; 1) anesthetics alone have either no or only a small effect on cytokine expression; 2) LPS provoked a large increase in microglia cytokine expression; 3) the inhaled anesthetics either had no effect on LPS-evoked responses or enhanced it; 4) propofol nearly eliminated the LPS pro-inflammatory cytokine response and improved cell survival as reflected by lactate dehydrogenase release. These data suggest that propofol may be a preferred anesthetic when it is desirable to minimize neuroinflammation.

## Introduction

Cognitive decline after major surgery and illness have been noticed and reported by patients and their families for decades. Older patients are particularly vulnerable to such an event, which has been coined post-operative cognitive dysfunction (POCD) [1]. Although many studies have been carried out in both humans and animal models, the mechanism of POCD remains unclear, although neuroinflammation has been implicated [2], [3]. Neuroinflammation has long been linked to the altered neural circuitry after trauma and in neurodegenerative diseases [4]–[6], suggesting that the activation of microglial cells may lead to changes in cognitive function through both direct and indirect effects on neurons. While surgery is most likely to trigger inflammation [2], [7]–[10], several lines of evidence have demonstrated that anesthetics are capable of modulating inflammation [11]. For example, in neuronal cell culture, the volatile anesthetic isoflurane increases levels of inflammatory mediators, which may contribute to tissue damage and neuronal death [12], while isoflurane down-regulates pro-inflammatory cytokines in peripheral neutrophils [13]. Moreover, anesthetics can reduce microglial activation [14]–[16] and the peripheral inflammatory response [17]–[20] to stimulants such as lipopolysaccharide (LPS) and interferon-gamma.

In this study, we will focus on the principle inflammatory cell in the central nervous system, the microglia, and examine the differential response of both resting and activated microglia when exposed to different anesthetics. These results will inform the clinical studies that ultimately will allow us to improve cognitive outcome in our older patient population.

## Materials and Methods

### Cell culture and treatment

The murine microglial BV-2 cell line [21], developed by Dr Blasi (University of Perugia, Perugia, Italy), was generously provided by Dr. Van Eldik at the University of Kentucky. BV-2 cells were cultured in Dulbecco's Modified Eagle's Medium/F12 supplemented with 10% fetal bovine serum, 100 IU/ml penicillin, 100 μg/ml streptomycin and 2 mM L-Glutamine. The cells were grown in a 37°C incubator, with humidified 95% air, 5% CO_2_. For experiments, serum-containing medium was removed and cells were treated with stimulus, anesthetics or vehicle in serum free medium. For the volatile anesthetic exposures, cells were placed in a tight gas chamber (Bellco Glass, Vineland, NJ) within the incubator and exposed to isoflurane (1.2%v/v, 2.4%v/v) (Phoenix Pharmaceuticals), sevoflurane (2%v/v, 4%v/v) (Abbott Laboratories), or air (air/5% CO2) for 6 h. The intravenous anesthetic, propofol (10 uM) (Sigma-Aldrich) was added to serum free medium with 0.1% DMSO (final concentration) and DMSO was added to the air controls (air/5% CO2) for 6 h as vehicle control (0.1% DMSO). The clinical formula for propofol was not used in this study as the emulsified lipid phase is unpredictably harmful to cell culture cells leading to irregular cellular effects. The lipid micelles, since they are not cleared as they would be in vivo, also “sequester” propofol, making it difficult to know what concentrations actually reach cells. By using DMSO, we can eliminate any potential effects of the lipid phase. Other cells were pretreated with 100 nM of lipopolysaccharide (LPS) for 1 h, followed by 6 h of incubation with the same anesthetic exposures. The supernatant and the BV-2 cell lysate were collected at the end of the 6 h anesthetic exposures.

### Cytokine Measurement

Aliquots of both supernatant and cell lysate were analyzed for inflammatory cytokines, with Luminex xMAP multiplexing technology [22] (Luminex Corp) in the Human Immunology Core at the University of Pennsylvania. Commercial MILLIPLEX® MAP kits (Millipore, Billerica, MA) were used in this study to simultaneously quantify the following cytokines: Interleukin-1β (IL-1β), IL-6, IL-10, and tumor necrosis factor α (TNF-α), using the Human Cytokines/Chemokines Panel 5 Plex kit (Millipore). The data (pg/ml) were normalized and expressed as pg cytokines per mg total protein (pg/mg).

### Cell survival assays

The lactate dehydrogenase (LDH) assay was used as a marker of cell injury after the anesthetic exposures using the LDH assay kit (Promegam Madison, WI), as described previously by Wei and colleagues [23]. Equal volumes of media and substrate (50 ul) were mixed together and incubated for 30 min at room temperature and then the reaction was stopped. The samples were quantified at 490 nm with a spectrophometric plate reader (OPSYS MRTM Absorbance Reader, Dynex Technologies, Chantilly, VA). The background signal from the media was measured and subtracted.

### Statistics

Statistics were performed using GraphPad Prism 5 software using unpaired t-tests or one-way analysis of variance (ANOVA) with multiple correction tests, as appropriate, and are detailed in the figure legends.

## Results

### Anesthetic exposures

**Figure 1:**
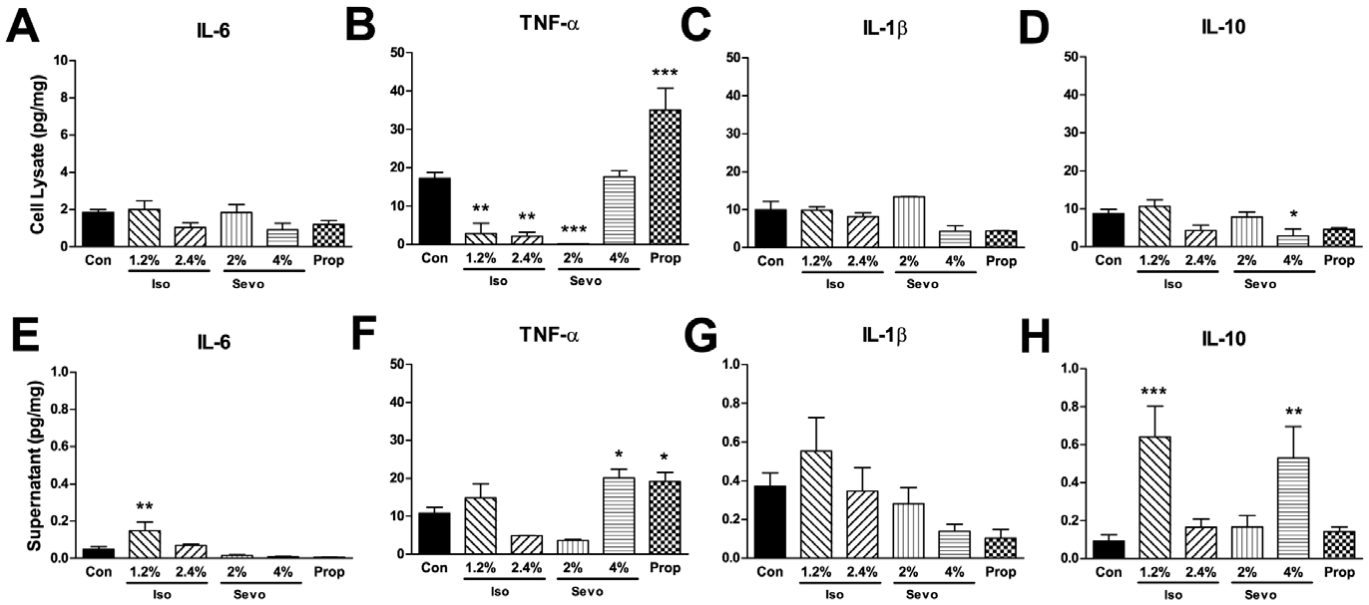
Anesthetics had minimal effects on the resting microglial cytokine response. The cytokine response to anesthetic exposures was evaluated in the cell lysate and supernatant fractions of cultured BV-2 microglial cells. In the cell lysates, no changes were found in IL-6 (A) or IL-1β (C) levels after the anesthetic exposures. However, TNF-α levels significantly declined after exposures to 1.2% and 2.4% isoflurane (Iso) and 2% sevoflurane (Sevo) but were significantly elevated after propofol (Prop) (B). IL-10 levels were significantly reduced only with 4% sevoflurane. In the supernatant, significant increases were found in both IL-6 after exposure to 1.2% isoflurane (E), and in TNF-α levels after 4% sevoflurane and propofol (F) and in IL-10 levels after 1.2% isoflurane and 4% sevoflurane (H). No changes were found in IL-1β levels (G). For all graphs, data are expressed as means ± SEM. For all anesthetics, n = 3 culture plates. No significant differences were found between the air controls and the air controls with DMSO and the control data were combined (n = 6). Significance was determined for both analyses by 1-way analysis of variance with Dunnett's multiple comparison test (control compared to all columns). *P<0.05, **P<0.01, ***P<0.001. SEM indicates standard error of the mean.

All three anesthetics had minimal to no effects on resting cytokine expression and release from microglia. In the cell lysates, modest but significant reductions in TNF-α were found after 1.2% and 2.4% isoflurane and 2% sevoflurane exposures and a small increase was observed after propofol exposure (Fig. 1B). No other significant effects were detected with IL-6, IL-1β or IL-10 expression (Fig. 1A, C, D), except for IL-10 after 4% sevoflurane (Fig. 1D). In the supernatant, modest but significant increases were found in IL-6 expression with 1.2% isoflurane (Fig. 1E), in TNF-α levels after 4% sevoflurane and propofol (Fig. 1F) and in IL-10 after 1.2% isoflurane and 4% sevoflurane (Fig. 1H). As in the cell lysate fraction, no significant changes were detected in the supernatant for IL-1β after any of the anesthetic exposures (Fig. 1G).

### LPS treatment before anesthetic exposures

**Figure 2:**
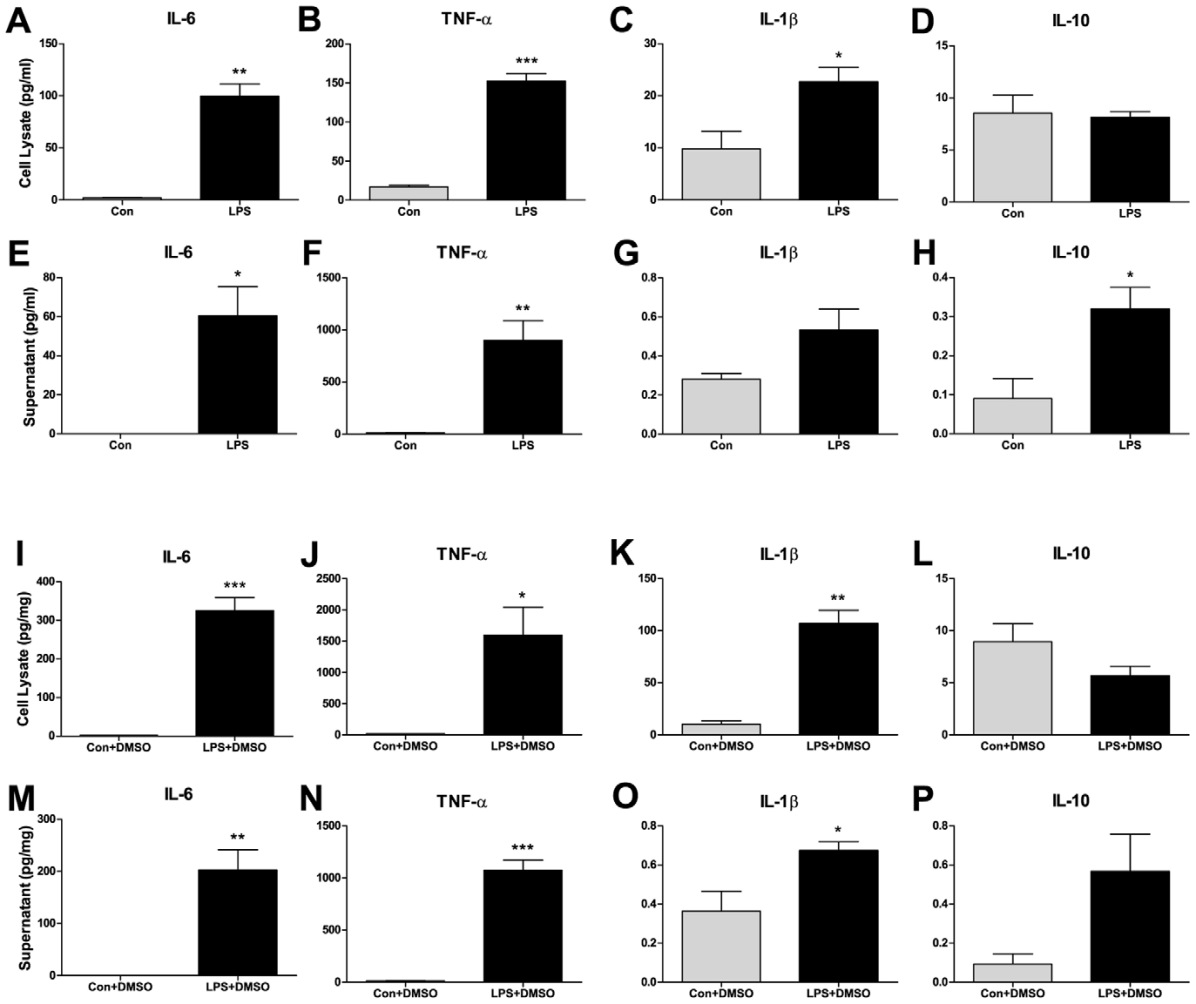
LPS stimulation increased the pro-inflammatory microglial cytokine response. The addition of LPS to BV-2 microglial cell cultures resulted in a significant increase in IL-6, TNF-α and IL-1β in the cell lysate fractions compared to air controls (A, B, C) and controls with DMSO (I, J, K). There was no change in IL-10 in the cell lysates without DMSO (D) or with DMSO (L). In the supernatant fractions, without DMSO, significant increases in IL-6, TNF-α and IL-10 (E, F, H) were found. Significant increases in IL-6, TNF-α and IL-1β were observed in supernatant fractions with DMSO (M, O, P). No significant differences in IL-1β levels after LPS alone (H) or in IL-10 levels after LPS with DMSO (P) were found. For all graphs, data are expressed as means ± SEM and n = 3 culture plates. Statistical significance was determined by unpaired t-tests. *P<0.05, **P<0.01, ***P<0.001. SEM indicates standard error of the mean.

LPS exposure produced significant increases in the expression of almost all of the pro-inflammatory cytokines, IL-6, TNF-α and IL-1β, in both the cell lysate and supernatant fractions compared to their air controls (Fig. 2A–C, E, F, H) or vehicle (DMSO) controls (Fig. 2 I–K, M–O) . The anti-inflammatory cytokine, IL-10, was unchanged with LPS stimulation, except in the supernatant fraction, compared to air controls (Fig. 2H).

**Figure 3:**
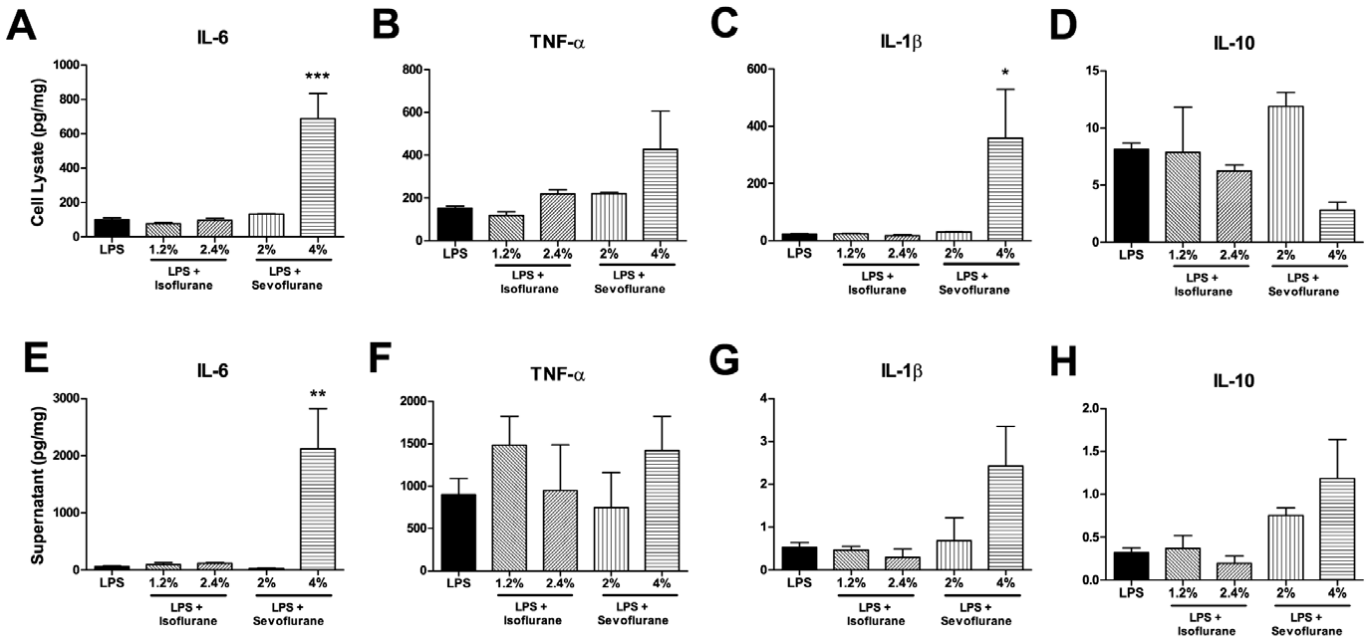
The inhaled anesthetics had minimal effects on the microglial cytokine response to LPS stimulation. Compared to LPS alone, the addition of volatile anesthetics significantly elevated the IL-6 (A) and IL-1β (C) response after exposure to both 4% sevoflurane and LPS. TNF-α (B) and IL-10 (D) levels were unchanged in the cell lysate fraction. In the supernatant fraction, only IL-6 (E) levels were significantly elevated after 4% sevoflurane and LPS exposure, while TNF-α (F), IL-1 β (G) and IL-10 (H) remained unchanged. For all graphs, data are expressed as means ± SEM and n = 3 culture plates. Significance was determined for both analyses by 1-way analysis of variance with Dunnett's multiple comparison test (control compared to all columns). *P<0.05, **P<0.01, ***P<0.001. SEM indicates standard error of the mean.

The inhaled anesthetics, isoflurane and sevoflurane, had a minimal effect on the response to LPS stimulation. For example, only 4% sevoflurane enhanced the IL-6 (Fig. 3A, 3E) and the IL-1 β (Fig. 3C) responses to LPS. No changes were observed for TNF-α (Fig. 3B, 3F) or IL-10 (Fig. 3D, 3H) in either fraction.

**Figure 4:**
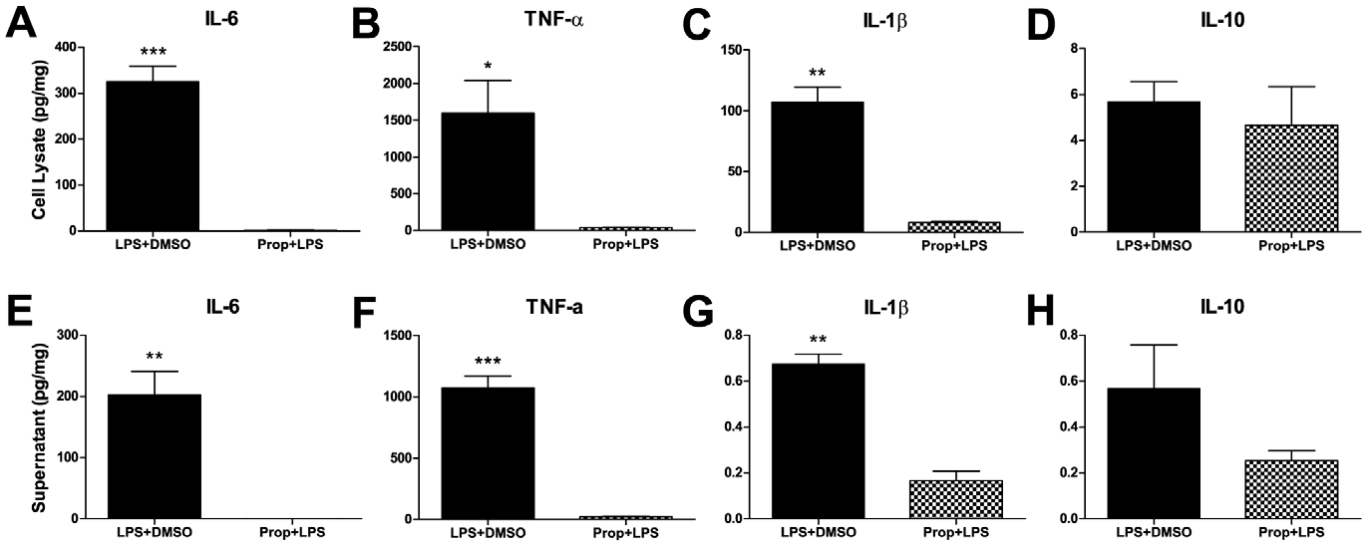
Propofol mitigated the effect of LPS pre-treatment. Cytokine levels are all significantly reduced with the addition of propofol compared to controls plus DMSO (vehicle) for IL-6 (A), TNF-α (B) and IL-1β (C) in both the cell lysate and supernatant fractions (E–G, respectively). There were no changes in the IL-10 levels after the addition of propofol to LPS in either the cell lysate (D) or supernatant (H) fractions. For all graphs, data are expressed as means ± SEM and n = 3 culture plates. Statistical significance was determined by unpaired t-tests. *P<0.05, **P<0.01, ***P<0.001. SEM indicates standard error of the mean.

Propofol, however, significantly mitigated the effect of LPS pre-treatment for the pro-inflammatory cytokines, IL-6, TNF-α and IL-1 β, in both the cell lysate (Fig. 4A–C) and supernatant (Fig. 4E–G). However, propofol did not significantly change the anti-inflammatory cytokine, IL-10, (Fig. 4D, 4H) after LPS stimulation.

### LDH Assay

**Figure 5:**
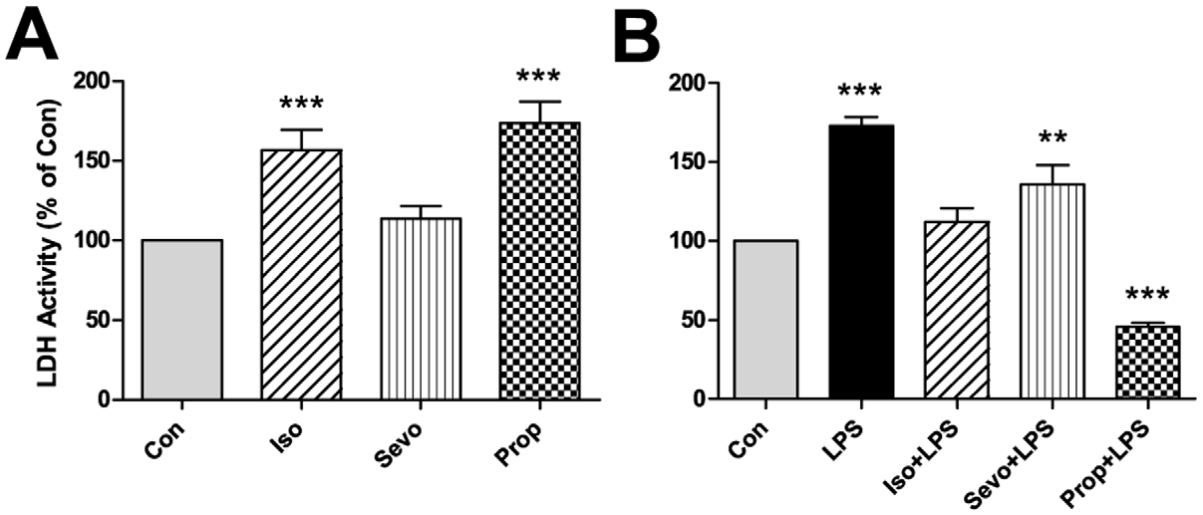
Propofol muted the LDH release after LPS pre-treatment. LDH release is significantly enhanced after isoflurane and propofol exposures (A). When microglial cultures are pre-treated with LPS, only propofol significantly reduces LDH release (B).

LDH assay was used to measure the adverse cellular response to both the anesthetic drugs alone and with LPS pre-treatment. The microglial cell cultures showed significant LDH release after both isoflurane and propofol exposures (Fig. 5A), while the LPS-stimulated LDH release was significantly inhibited by the addition of propofol (Fig. 5B).

## Discussion

In this study, we sought to examine the effect of clinical anesthetics on microglia reactivity under physiological and stressed conditions. We found that both the intravenous anesthetic, propofol, and the inhaled anesthetics, isoflurane and sevoflurane, elicit only modest changes in cytokine expression in unstimulated, resting microglia. As expected, LPS elicits a robust cytokine response, which was essentially unaltered by subsequent sevoflurane or isoflurane treatment. The most striking observation of this study is that propofol almost completely mitigates the LPS response of microglia, and also protects them from LPS-induced LDH release.

### Microglial model

In patients with neurodegenerative disease, the CNS has increased numbers of activated microglia and astrocytes, and elevated pro-inflammatory protein levels, such as IL-6 and TNF-α [24]. The microglia in such brain tissue is therefore considered “primed” [25] and poised to respond in an exaggerated manner with further stimulation. Cell culture in general, and cell-lines specifically, have limited translatability to more integrated in vivo systems, but this LPS-stimulated microglial model, allows us to study the incremental effect of clinically used drugs, like the anesthetics [26], directly on microglia and without the in vivo complexity that make direct contributions difficult to evaluate.

### Volatile Anesthetics

Even though both isoflurane and sevoflurane have been associated with neuronal injury, apoptosis and cognitive problems in pre-clinical studies [27], [28], they seem to have minimal effect in our highly focused neuroinflammation model, and could therefore be considered “permissive.” However, others have reported that pretreatment, or delayed treatment with isoflurane reduced LPS and interferon-gamma induced microglial cell injury [14], [15], [20] and down-regulated peripheral pro-inflammatory cytokines after zymosan induced peritonitis [13]. These studies suggest dose and timing of treatment also play important roles on outcome. Other studies of this question are inconsistent. For example, isoflurane alone increased pro-inflammatory cytokines in the mouse brain [12] and was found to be more neurotoxic [29], [30] than most other inhaled agents, although reports of rigorous side-by-side comparisons have been rare.

### Propofol

We found that propofol exhibits very robust anti-inflammatory effects that reduce levels of TNFa, IL-6 and IL-1b expression and in LPS stimulated microglia, almost to baseline. Isoflurane and propofol appear to have small effects on LDH release of their own but, when combined with LPS pretreatment, we found that the toxic effect was significantly reduced – but only for propofol. The latter finding is easy to interpret in the context of the cytokine data discussed above. The former result, however, we have no good explanation for at this point, but it clearly suggests competing pathways for propofol that are modulated by the LPS response. The mechanism underlying this prominent anti-inflammatory effect by propofol may reside in an inhibition of the p38 pathway [31], downregulation of TLR4, as well as elevation of glycogen synthase kinase (GSK) 3b [16]. A similar protective role of propofol has been recently shown using concentrations of propofol considerably higher than achieved clinically, and without comparison to other anesthetics [16]. Moreover, propofol has been shown to reduce neuroapoptosis caused by isoflurane [30] in mice. But the mechanism could also be as simple as radical scavenging. Translation to clinical studies has been inconsistent. For example, coronary artery bypass graft (CABG) patients had better cognitive performance after sevoflurane based anesthesia as compared to propofol [32], while other clinical studies have shown that most general anesthetics, including propofol, are associated with POCD, perhaps less with desflurane [33]–[37], although again, timing and definition of POCD varies widely.

In summary, general anesthetics in clinically applied concentrations had only modest effects on microglia responses in the absence of “priming”. However, LPS stimulation segregates the anesthetics. While the inhaled anesthetics allowed the LPS response to proceed unabated, propofol dramatically reduced the response. These results suggest that propofol may be a preferred general anesthetic when perioperative goals include a muted neuroinflammatory response.
